# Steroid sulfatase-deficient mice exhibit endophenotypes relevant to Attention Deficit Hyperactivity Disorder

**DOI:** 10.1016/j.psyneuen.2011.06.006

**Published:** 2012-02

**Authors:** Simon Trent, Alison Dennehy, Heather Richardson, Obah A. Ojarikre, Paul S. Burgoyne, Trevor Humby, William Davies

**Affiliations:** a Behavioural Genetics Group, Schools of Psychology and Medicine, Cardiff University, Cardiff, UK; b School of Psychology, Cardiff University, Cardiff, UK; c MRC Centre for Neuropsychiatric Genetics and Genomics and Department of Psychological Medicine and Neurology, School of Medicine, Cardiff University, Cardiff, UK; d Neuroscience and Mental Health Research Institute, Cardiff University, Cardiff, UK; e MRC National Institute for Medical Research, London, UK

**Keywords:** Anxiety, Basal forebrain, COUMATE, Dehydroepiandrosterone sulfate, Locomotor activity, Neurosteroid

## Abstract

Attention Deficit Hyperactivity Disorder (ADHD) is a common neurodevelopmental condition characterised by inattention, impulsivity and hyperactivity; it is frequently co-morbid with anxiety and conduct disorders, sleep perturbation and abnormal consummatory behaviours. Recent studies have implicated the neurosteroid-modulating enzyme steroid sulfatase (STS) as a modulator of ADHD-related endophenotypes. The effects of steroid sulfatase deficiency on homecage activity, feeding/drinking behaviours, anxiety-related behaviours (assayed in light-dark box and open field paradigms), social dominance and serum steroid hormone levels were determined by comparing 40,XY and 39,X^Y*^O mice. Subsequently, mice administered the steroid sulfatase inhibitor COUMATE acutely were compared to vehicle-treated mice on behavioural tasks sensitive to enzyme deficiency to dissociate between its developmental and ongoing effects. 39,X^Y*^O mice exhibited heightened reactivity to a novel environment, hyperactivity in the active phase, and increased water (but not food) consumption relative to 40,XY mice during a 24 h period; the former group also demonstrated evidence for heightened emotional reactivity. There was no difference in social dominance between the 40,XY and 39,X^Y*^O mice. COUMATE administration had no effect on homecage activity, water consumption or anxiety measures in the open field. 39,X^Y*^O mice exhibited significantly lower dehydroepiandrosterone (DHEA) serum levels than 40,XY mice, but equivalent corticosterone levels. Together with previous data, the present results suggest that steroid sulfatase may influence core and associated ADHD behavioural endophenotypes via both developmental and ongoing mechanisms, and that the 39,X^Y*^O model may represent a useful tool for elucidating the neurobiological basis of these endophenotypes.

## 1 Introduction

Attention Deficit Hyperactivity Disorder (ADHD) is a common neurodevelopmental disorder characterised by inattention, impulsivity and hyperactivity and affecting 3–7% of the school-age population (Faraone et al., 2003, Thapar et al., 2005). In a significant proportion of cases, ADHD symptoms may persist into adulthood with adverse consequences for employability, social interactions, and substance abuse (Able et al., 2007). ADHD is often co-morbid with other disabling conditions including conduct disorder (typified by heightened aggression) and anxiety disorders (Cormier, 2008).

As ADHD is diagnosed between 4 and 6 times more frequently in boys than in girls, sex-linked genes have been implicated as modulators of risk (Holden, 2005). Based on work in mouse models, the *STS* gene (which encodes the enzyme steroid sulfatase) was proposed as a novel ADHD candidate (Davies et al., 2007); in mice *Sts* is located within the pseudoautosomal region of the sex chromosomes, whereas its human orthologue resides within the X-specific region at Xp22.3. The function of steroid sulfatase is to cleave sulfate groups from steroid hormones (including dehydroepiandrosterone sulfate, DHEAS) in order to alter their biological activity (Reed et al., 2005); in the brain, sulfated and non-sulfated steroids can influence the function of GABA_A_ and NMDA receptors, and may exert distinct effects (Compagnone and Mellon, 2000). Both DHEAS, and its non-sulfated form DHEA, are negative modulators of the GABA_A_ receptor, and positive modulators of the NMDA receptor (Yadid et al., 2010).

Work in humans has shown that individuals with deletions encompassing *STS* (or with inactivating mutations within the gene) are at elevated risk of developing ADHD (Kent et al., 2008) and that *STS* is expressed in regions of the developing brain relevant to ADHD pathology (Stergiakouli et al., 2011). Moreover, *STS* polymorphisms are associated with: (i) vulnerability to developing the disorder (Brookes et al., 2008), (ii) cognitive function in ADHD (Stergiakouli et al., 2011) and (iii) altered gene expression in the healthy brain (Brookes et al., 2010). Finally, it has previously been suggested that levels of systemic DHEA (the non-sulfated form of DHEAS), but not another steroid hormone (cortisol), are lower in individuals with ADHD (Wang et al., 2011b) and that methylphenidate may partially exert its therapeutic effect in ADHD subjects by normalising DHEA levels (Maayan et al., 2003, Wang et al., 2011a).

Through comparing wildtype to mutant 39,X^Y*^O mice (which lack the *Sts* gene but no other known genes as a consequence of end-to-end fusion of the X and Y chromosomes (Odorisio et al., 1998)), we have shown that *Sts* gene-deletion results in impaired attention; inhibition of steroid sulfatase in wildtype male mice with the specific inhibitor COUMATE recapitulated this deficit, suggesting that the enzyme was influencing attention via its ongoing function (Davies et al., 2009). We also reported that neither *Sts* deletion, nor COUMATE administration, appeared to influence locomotor activity. However, our previous analysis was sub-optimal in that activity was assayed using a crude measure (beam breaks) over a short timeframe (1 h) during the day (i.e. non-active phase), and in a different environment to the homecage of the mice (Davies et al., 2009). Here, we assessed locomotor activity in 39,X^Y*^O and wildtype mice in a more sophisticated manner by continuously tracking individuals’ movements in a homecage-like environment over an extended period. ADHD subjects have been reported to show altered novelty-seeking behaviour (Cho et al., 2009, Purper-Ouakil et al., 2010), perturbed circadian rhythm and sleep disorders (Walters et al., 2008, Chiang et al., 2010), and abnormalities in consummatory behaviours (Cortese et al., 2007, Quinn, 2008). Our homecage experiment also enabled us to assay reactivity to a novel environment, provided a crude index of the sleep-wake cycle and allowed us to measure patterns of food and water consumption. As anxiety disorders are comorbid with ADHD in up to 40% of cases (Spencer, 2006), we also sought to compare anxiety-related behaviours in 39,X^Y*^O and wildtype mice using two well-defined behavioural paradigms: the light-dark box and the open field (Ramos, 2008, Milner and Crabbe, 2008). Finally, given previous evidence for heightened aggression in 39,X^Y*^O mice (Davies et al., 2009), and suggestions that ADHD schoolchildren are both more likely to be bullies, and to be bullied themselves (Holmberg and Hjern, 2008), we directly compared social dominance in the two groups using the tube test (Lindzey et al., 1961). Aggression is often employed in social animals such as mice as a mechanism through which to maintain social hierarchies (Miczek et al., 2004). As in our previous study (Davies et al., 2009), to ascertain whether behavioural differences between *Sts* gene-deletion and wildtype mice were due to the developmental or ongoing effects of steroid sulfatase loss, we administered the specific steroid sulfatase inhibitor COUMATE to wildtype mice; this drug has been shown to attenuate brain steroid sulfatase activity by ∼70% 24 h after administration (Nicolas et al., 2001). Finally, given the results of steroid hormone analyses in subjects with ADHD and neurotypical controls, we assayed systemic levels of DHEA and corticosterone (the main glucocorticoid in rodents) in our wildtype and mutant mice.

## 2 Methods

### 2.1 Subjects for behavioural experiments

39,X^Y*^O mutant and 40,XY wildtype mice were bred at MRC National Institute for Medical Research (NIMR), London. 39,X^Y*^O mice were produced from three crosses: (i) 39,X^*Paf*^O x 40,XY*, (ii) 39,X^*Paf*^O x 39,X^Y*^O, and (iii) 40,In(X)^*Paf*^/X x 40,XY*. *Paf* is an X-linked mutation involving a small inversion spanning the pseudoautosomal boundary which gives rise to a ‘patchy fur’ phenotype in heterozygous females and males (Korobova et al., 1998), In(X) is an X chromosome containing a large paracentric inversion (Evans and Phillips, 1975) and Y* is a Y chromosome that has been hijacked by a non-Y centromere attached distal to the pseudoautosomal region and lacks *Sts*; recombination between Y* and the X generates the X^Y*^ chromosome that also lacks *Sts* (Eicher et al., 1991, Burgoyne et al., 1998). 39,X^Y*^O males could be identified though a combination of checking gonadal type, checking for the presence of patchy fur between postnatal days 7–10, and polymerase chain reaction (PCR) for the *Sts* gene from tail biopsy at weaning (primer sequences: 5′-GCTCGCTGACATCATCCTC-3′ and 5′-CACCGATGCCCAGGTCGTC-3′; for detailed PCR methods see Davies et al., 2009). 40,XY wildtype males were generated from 40,XY × 40,XX crosses. Care was taken to keep the genetic backgrounds of the crosses equivalent (i.e. predominantly that of the MF1 albino strain (NIMR colony)) but with two C3H strain-derived factors which enable fertility of 39,X^Y*^O males; also, the 40,XY males carry a Y chromosome derived from the LT strain from which the Y* chromosome originated. 36 40,XY wildtype mice and 19 39,X^Y*^O mice were transferred to Cardiff University for behavioural testing. 48 male MF1 mice (∼3 months) were obtained from Harlan, UK for the pharmacological assays.

### 2.2 Animal husbandry

In Cardiff, mice from NIMR were treated with Baytril and Norodine-24 antibiotics for one month in a negative-pressure isolator to cure a *Pasteurella pneumotropica* infection prior to release onto the open racks. These mice were then housed in a holding room maintained at 21 ± 2 °C and 50 ± 10% humidity, with a 12 h light-dark cycle (lights on at 0700 h), either singly (due to the tendency of 39,X^Y*^O mice to fight) or in groups of up to three with mice of the same karyotype. Behavioural testing in 40,XY and 39,X^Y*^O mice took place between the ages of 3–9 months. Mice for the pharmacological study were housed in the same holding room (4 mice per cage) and were tested between the ages of 3–6 months. Mice were allowed *ad libitum* access to food and water throughout behavioural testing. Animals were treated in accordance with the Animal (Scientific Procedures) Act (United Kingdom, 1986). All efforts were made to minimize animal suffering, to reduce the number of animals used, and to utilize alternatives to *in vivo* techniques, if available.

### 2.3 Drug administration

Mice were administered either vehicle (0.5% methylcellulose, 0.9% NaCl in distilled water, PO) or COUMATE (10 mg/kg in the same vehicle PO, COUMATE synthesised according to Nicolas et al., 2001) 24 h prior to behavioural testing.

### 2.4 Homecage behavioural analysis

39,X^Y*^O (*n* = 19) and 40,XY mice (*n* = 25) were tested using a battery of ‘Phenotyper’ homecages (Noldus Information Technology) (30 cm × 30 cm × 35 cm (height)) for a 24 h period coincident with one light-dark cycle in the holding room (lights on for 12 h, lights off for 12 h), with behavioural testing starting between 0700 h and 1000 h. Mice were housed singly for the testing period, and the karyotypes were pseudorandomly assigned to the different homecages to negate any systematic order or homecage effects. Each homecage contained a perspex shelter, a running wheel, a water bottle connected to a lickometer and a food hopper, and was lined with clean absorbent black paper (Supplementary Fig. S1A). The top-unit of each cage housed an infra-red sensitive camera to continuously record the position of the mouse and an infra-red light source providing constant, even, illumination of the cage floor. Tracking and data recording was performed using EthoVision XT software (Noldus Information Technology) running on a high specification personal computer (Dell, UK). Data were recorded for 8 bins, each of 3 h duration. The tracking arena was sub-divided into 5 zones: running wheel, shelter, food zone, drinking zone and arena floor (Supplementary Fig. S1B). The main output measures were: total distance travelled in the tracking arena, time spent in each zone, number of rotations of the running wheel, and number of licks of the water bottle spout. At the end of the test session, total volumes of water and weight of food consumed were measured. The apparatus was thoroughly cleaned with 1% acetic acid between mice.

Behaviourally naïve mice for the pharmacological study (Vehicle: *n* = 12, COUMATE: *n* = 12) were tested in the same homecages, using a modified protocol: these mice were run in the light for 3 h, and in the dark for 12 h, with testing commencing between 1600 and 1700 h. This protocol ensured that mice had opportunity to habituate to the apparatus, and that the onset of the dark phase occurred 24 h after vehicle/COUMATE administration. Data was recorded for 15 bins, each of 1 h duration. Identical output measures were recorded as for the genetic study. The apparatus was thoroughly cleaned with 1% acetic acid between mice.

### 2.5 Anxiety tests

After homecage monitoring, 39,X^Y*^O (*n* = 19) and 40,XY mice (*n* = 25) were tested in the light-dark box followed, five days later, by testing in the open field. Behaviourally naïve MF1 male mice for the pharmacological study (Vehicle: *n* = 12, COUMATE: *n* = 12) were tested on the open field only.

### 2.6 Light-dark box

The apparatus consisted of two adjacent perspex boxes (one black and one white) of equal size (30 cm × 30 cm × 30 cm), connected by a hole (7 cm × 7 cm). The black (dark) box was covered with a black perspex lid, whilst the white (light) box was illuminated from above at 300 lux. Mice were placed at the far end of the light box, and allowed to explore freely for 5 min. Test sessions were recorded to videotape via a camera directly above the apparatus for later coding by researchers blind to karyotype. The main behavioural measures recorded were: latency to enter the dark box, time spent in each box, number of transitions between the two boxes, number of fecal boli deposited, and presence/absence of urine. Mice were scored as being in a box once all four legs had entered the box. The apparatus was thoroughly cleaned with 1% acetic acid between mice. Mutant and control mice were run in a pseudorandomised order between 1200 h and 1700 h.

### 2.7 Open field

The apparatus consisted of a large square perspex box with a black floor and white sides of size (75 cm × 75 cm × 45 cm (height)), illuminated from above at 300 lux. Mice were placed in one corner, and allowed to explore freely for 5 min. Test sessions were recorded to videotape via a camera directly above the apparatus for later coding by researchers blind to karyotype/drug treatment. During videotape analysis, the arena was subdivided into quadrants, and into an outer and an inner zone (60 cm × 60 cm). The main behavioural measures recorded were: latency to enter the inner zone, time spent in each zone, number of quadrants entered, number of rears, number of fecal boli deposited, and presence/absence of urine. Mice were scored as being in a zone once all four legs had entered the zone. The apparatus was thoroughly cleaned with 1% acetic acid between mice. Mutant and control mice were run in a pseudorandomised order between 1030 h and 1600 h.

### 2.8 Social dominance tube test

One 39,X^Y*^O mouse and one 40,XY mouse (matched as closely as possible for weight) were placed head first at opposite ends of a clear plastic tube (33 mm internal diameter, 30 cm length) and released simultaneously. The bout ended when one mouse withdrew completely from the tube. The mouse remaining in the tube was designated the winner, and the retreating mouse the loser. Each pairing had three bouts, and there were 19 separate pairings (57 bouts in total). The test was run between 1400 h and 1700 h.

### 2.9 Steroid hormone analysis

Trunk blood was obtained between 1100 h and 1430 h from adult (8–10 months) 40,XY (*n* = 7) and 39,X^Y*^O (*n* = 6) mice generated using the crosses described above and group-housed in the Animal Unit at MRC National Institute for Medical Research. Blood was collected in Microtainer Gold tubes (BD Biosciences). Tubes were inverted 5 times, and left for ∼30 min for clotting to occur. Serum was separated by centrifugation at 14,000 rpm for 2 min, aliquoted into eppendorfs and stored at −80 °C. Steroid hormone levels were assayed from serum samples using ELISA kits (dehydroepiandrosterone (DHEA), DRG International; corticosterone, Enzo Biosciences) according to the manufacturer's instructions, with sample dilution as necessary and samples run in triplicate (DHEA) or duplicate (corticosterone). For the DHEA ELISA, cross reactivity was 100% for DHEA and <0.072% for other related compounds; analytical sensitivity of the assay was 0.108 ng/ml. For the corticosterone ELISA, cross reactivity was 100% for corticosterone, 28.6% for deoxycorticosterone and <2% for other related compounds; analytical sensitivity of the assay was 26.99 pg/ml. Standard curves were determined using SigmaPlot 11.0 (Systat Software Inc.) according to the hyperbolic decay curve defined by the following equation: *y* = *y*_0_ + (*ab*/(*b* + *x*)) where *y*_0_, *a* and *b* are constants. Sample outliers (i.e. values >2 standard deviations below or above the group mean) were excluded from the analysis.

### 2.10 Statistics

Statistics were analysed using SPSS 16.0 (IBM Corporation, New York). Normal data were analysed by unpaired two-tailed *t*-test/Two Way Repeated Measures ANOVA, with factors of karyotype/drug treatment and timepoint. Skewed data were transformed before parametric analysis as appropriate. Where sphericity assumptions were violated in Two Way ANOVA, Greenhouse-Geisser corrected degrees of freedom values are presented. When ANOVA indicated an interaction between karyotype/drug treatment and TIMEPOINT, *post hoc* pairwise comparisons were performed using unpaired *t*-tests between each datapoint. Non-parametric data were analysed by two-tailed Mann–Whitney *U* test. Frequency data were analysed by two-tailed Fisher Exact Test or *χ*^2^ analysis. *p*-Values of ≤0.05 were regarded as significant; for *post hoc* tests *p*-values were adjusted to account for multiple testing. Data are reported as mean values ± standard error of the mean. Where appropriate, effect sizes were calculated according to Cohen (1988) and *d* values are presented. No differences were observed between 39,X^Y*^O mice with 39,X^*Paf*^O or 40,In(X)^*Paf*^/X mothers (*n* = 14 and *n* = 5 respectively), or between singly and group-housed 39,X^Y*^O mice (*n* = 12 and *n* = 7 respectively) unless stated in the text.

## 3 Results

### 3.1 39,X^Y*^O mice are hyperactive and consume more water (but not food) relative to 40,XY mice

**Figure 1:**
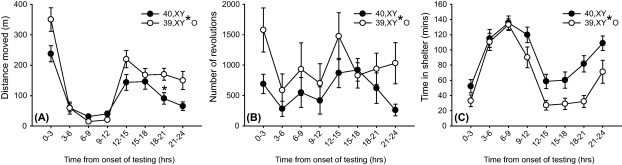
Behaviour of 40,XY (*n* = 25) and 39,X^Y*^O (*n* = 19) mice in automated homecages over a 24 h period (lights off after 12 h). (A) 39,X^Y*^O mice showed heightened reactivity to the novel environment (0–3 h), and increased activity during the dark phase (12–24 h) relative to 40,XY mice as indexed by lateral activity. *Corrected *p*-value <0.05 for *post hoc* comparison. (B) 39,X^Y*^O mice made significantly more revolutions in the homecage running wheels throughout the testing period relative to 40,XY mice. (C) 39,X^Y*^O mice spent significantly less time in the shelter throughout the testing period than 40,XY mice.

Individual mouse tracking data showed that 39,X^Y*^O mice were more hyperactive than 40,XY mice during the initial 3 h of homecage testing but not during the remainder of the light phase; throughout the dark phase, 39,X^Y*^O mice were ∼70% more active than 40,XY mice (Cohen's *d* = 0.86) (effect of karyotype: *F*[1,42] = 5.92, *p* < 0.05, effect of karyotype × timepoint: *F*[3.87,42] = 4.00, *p* < 0.005, Fig. 1A). Conservative *post hoc* testing revealed a significant difference between karyotypes 18–21 h after the onset of testing (corrected *p*-value < 0.05). Analysis of a second, more variable, measure of activity (number of running wheel revolutions) also revealed 39,X^Y*^O mice to be more active than their wildtype counterparts (Cohen's *d* = 0.64) (effect of karyotype: *F*[1,42] = 4.66, *p* < 0.05, Fig. 1B). 39,X^Y*^O mice spent significantly less time in the shelter than 40,XY mice across all time bins (Cohen's *d* = 0.97) (effect of karyotype: *F*[1,42] = 9.41, *p* < 0.005, Fig. 1C).

39,X^Y*^O mice drank a significantly greater volume of water per unit bodyweight than 40,XY mice (0.22 ± 0.01 ml/g vs. 0.18 ± 0.01 ml/g respectively, *t*[42] = −2.62, *p* < 0.05, Cohen's *d* = 0.81). This is consistent with a strong trend towards a greater degree of licking behaviour in 39,X^Y*^O mice (total licks: 4795 ± 1292 vs. 2005 ± 551, *t*[24.5] = −1.96, *p* = 0.06) and with these mice spending significantly more time in the drinking zone (124 ± 33 s vs. 37 ± 12 s, *t*[20.4] = −3.17, *p* < 0.005, Cohen's *d* = 0.98). In contrast, there was no difference between 39,X^Y*^O and 40,XY mice with respect to overall food consumption per unit bodyweight (0.20 ± 0.01 g/g vs. 0.24 ± 0.04 g/g respectively, *t*[27.7] = 1.00, *p* = 0.33), despite 39,X^Y*^O spending more time in the feeding zone (data not shown). In some studies, food/water consumption are normalised to bodyweight^0.75^ to account for metabolic scaling (e.g. Doe et al., 2009); this alternative analysis did not alter the pattern of data (effect of karyotype on water consumption: *t*[42] = −2.30, *p* < 0.05, effect of karyotype on food consumption: *t*[27.5] = 1.14, *p* = 0.27). Drinking and feeding patterns were similar between the two groups, with the majority of consummatory behaviours occurring from the onset to the end of the dark phase (data not shown).

### 3.2 39,X^Y*^O mice differ from 40,XY mice on aspects of anxiety-related behaviour

In the light-dark box, 39,X^Y*^O mice initially entered the dark box from the light box significantly more rapidly than wildtype mice (Cohen's *d* = 0.67), and defecated/urinated to a significantly greater extent (Table 1); singly housed 39,X^Y*^O mice urinated more frequently than group-housed 39,X^Y*^O mice (Fisher Exact Test, *p* < 0.05). 40,XY and 39,X^Y*^O mice did not differ with respect to a further measure of anxiety-related behaviour i.e. time spent in the light box, nor with respect to a measure of in-test activity, the number of transitions between boxes (Table 1).

**Table 1:** Anxiety-like profile in 40,XY and 39,X^Y*^O mice.

Test and measure	40,XY (*n* = 25)	39,X^Y*^O (*n* = 19)	Significance level
*Light-dark box*			
Latency to enter dark box (s)	47.8 ± 7.8	28.3 ± 4.5	*t*[37.4] = 2.17, *p* < 0.05
Time spent in light box (s)	113.6 ± 11.4	114.1 ± 21.8	*t*[27.6] = −0.02, *p* = 0.99
Transitions between boxes	9.0 ± 1.3	8.8 ± 1.7	*t*[42] = 0.06, *p* = 0.96
Fecal boli deposited	0.1 ± 0.1	1.0 ± 0.3	*U* = 139, *p* < 0.005
Mice urinating/mice not urinating	0/25	6/13	Fisher Exact Test, *p* < 0.005
*Open field*			
Latency to enter inner zone (s)	86.0 ± 21.0	133.1 ± 27.0	*t*[42] = −1.40, *p* = 0.17
Time spent in outer zone (s)	253.3 ± 7.1	274.2 ± 5.6	*t*[42] = −2.20, *p* < 0.05
Transitions between quadrants	36.0 ± 4.4	32.4 ± 4.2	*t*[42] = 0.57, *p* = 0.57
Rears	14.7 ± 2.6	13.2 ± 3.1	*t*[42] = 0.38, *p* = 0.71
Fecal boli deposited	1.9 ± 0.4	3.1 ± 0.5	*U* = 159.5, *p* = 0.06
Mice urinating/mice not urinating	2/23	4/15	Fisher Exact Test, *p* = 0.38

40,XY and 39,X^Y*^O mice differed on the main measure of anxiety-related behaviour in the open field, whereby the latter group spent a significantly greater amount of time in the outer zone (Cohen's *d* = 0.68); 39,X^Y*^O mice also took longer to enter the inner zone from the outer zone (Table 1). 39,X^Y*^O mice showed a strong, but non-significant, trend towards increased defecation in this test, and an increased rate of urination (Table 1). There was no significant difference between the two groups with respect to measures of activity or exploration in the open field test (Table 1).

### 3.3 39,X^Y*^O and 40,XY mice do not differ in their social dominance

Of 57 bouts in the tube test, 40,XY mice won 31, and 39,X^Y*^O mice won 26 (*χ*^2^[1] = 0.28, *p* = 0.60). Heavier mice did not win significantly more frequently than lighter mice irrespective of genotype (*χ*^2^[1] = 0.28, *p* = 0.60); therefore, the fact that 39,X^Y*^O mice were lighter than 40,XY mice (41.2 ± 0.7 g vs. 44.6 ± 0.4 g) is unlikely to confound the main result.

### 3.4 COUMATE-treated mice do not differ from vehicle-treated mice with respect to locomotor activity, water consumption or anxiety-related measures

Although both vehicle and COUMATE-treated groups showed the expected patterns of lateral activity (effect of timepoint, *F*[4.2,92.8] = 21.15, *p* < 0.001), there was no effect of drug treatment (*F*[1,22] = 0.08, *p* = 0.77), nor any interaction between drug treatment and timepoint (*F*[4.2,92.8] = 0.41, *p* = 0.81). There was also no difference in the total number of running wheel revolutions between COUMATE and vehicle-treated mice (8730 ± 1228 vs. 9864 ± 1945 respectively, *t*[22] = 0.49, *p* = 0.63). During homecage behavioural testing, COUMATE-treated mice did not consume significantly more water per unit bodyweight than vehicle-treated mice (0.18 ± 0.03 ml/g vs. 0.16 ± 0.02 ml/g respectively, *t*[22] = −0.40, *p* = 0.69), and nor did they make significantly more licks (1176 ± 377 vs. 649 ± 122 respectively, *t*[22] = −1.38, *p* = 0.18). Vehicle and COUMATE-treated mice did not differ with respect to anxiety-related behavioural measures in the open field test (Table 2).

**Table 2:** Behavioural measures in the open field in mice treated with vehicle or the steroid sulfatase inhibitor COUMATE.

Test and measure	Vehicle-treated (*n* = 12)	COUMATE-treated (*n* = 12)	Significance level
*Open field*			
Latency to enter inner zone (s)	37.3 ± 7.2	43.7 ± 14.2	*t*[22] = −0.40, *p* = 0.69
Time spent in outer zone (s)	224.3 ± 7.4	234.9 ± 6.8	*t*[22] = −1.06, *p* = 0.30
Transitions between quadrants	45.9 ± 3.4	41.1 ± 4.4	*t*[22] = 0.87, *p* = 0.39
Rears	40.8 ± 3.4	29.8 ± 3.5	*t*[22] = 2.29, *p* < 0.05
Fecal boli deposited	1.7 ± 0.5	2.1 ± 0.5	*U* = 62.5, *p* = 0.59
Mice urinating/mice not urinating	2/10	2/10	Fisher Exact Test, *p* = 1.0

### 3.5 39,X^Y*^O mice exhibit lower levels of serum DHEA, but equivalent levels of corticosterone, to 40,XY mice

**Figure 2:**
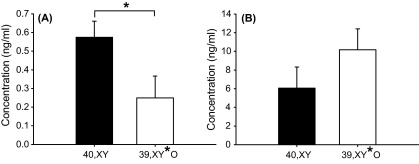
DHEA (A) and corticosterone (B) levels in serum of adult 40,XY (*n* = 6) and 39,X^Y*^O (*n* = 6) mice (*p < 0.05).

One outlier 40,XY mouse was excluded from each analysis. Serum DHEA levels were significantly lower in 39,X^Y*^O mice than 40,XY mice (*t*[10] = 2.25, *p* < 0.05, Cohen's *d* = 1.42), whilst corticosterone levels did not differ between the two groups (*t*[10] = −1.29, *p* = 0.23) (Fig. 2).

## 4 Discussion

This study investigated whether steroid sulfatase deficiency could influence behavioural endophenotypes associated with ADHD. The main finding was that 39,X^Y*^O mice were hyperactive relative to 40,XY mice upon exposure to a novel environment and during the active phase. Consistent with our previous data (Davies et al., 2009), 39,X^Y*^O and 40,XY mice were found to be equally active for the majority of the light phase. Importantly, given that isolation rearing may elicit hyperlocomotion in adult mice (Koike et al., 2009), we found no difference in activity profiles between singly and group-housed 39,X^Y*^O mice (Supplementary Fig. S2). It has previously been reported that DHEA levels are lower in individuals with ADHD than in neurotypical subjects, and that serum DHEA(S) levels correlate inversely with the hyperactivity subscale in ADHD patients (Strous et al., 2001, Wang et al., 2011b). Therefore, we analysed steroid hormone serum levels in 39,X^Y*^O and 40,XY mice. We found that levels of DHEA (but not corticosterone) were lower in the former group; these data suggest some overlap between the physiology of ADHD subjects and 39,X^Y*^O mice, and support the concept that low levels of systemic DHEA may be associated with increased activity (Supplementary Fig. S3).

We also found that 39,X^Y*^O mice consumed a greater volume of water (but not food) during testing than 40,XY mice; this dissociation suggests that the greater degree of water consumption in 39,X^Y*^O mice may not simply be a function of their greater locomotor activity. Interestingly, ADHD subjects have been reported to drink significantly more water than typically developing individuals in a free-drinking experiment, possibly as an osmo-regulatory mechanism (Oades et al., 1998). The general patterns of activity and consummatory behaviours in 39,X^Y*^O and 40,XY mice over a 24 h period were similar, indicating that there were no gross disturbances in the sleep-wake cycle of the former group. However, more detailed assessment of circadian rhythms in these mice might be performed over longer testing periods in the future.

Data from two tasks assaying aspects of anxiety provided converging evidence for enhanced anxiety-related behaviours in 39,X^Y*^O mice relative to 40,XY mice. These data are likely to specifically reflect group differences in anxiety *per se* because: (i) in-test measures of activity/exploration did not differ between groups, (ii) the groups showed equivalent homecage activity during the light phase (when tests were performed), (iii) food/water consumption was equivalent between groups during the light phase and (iv) most measures were indifferent to whether the 39,X^Y*^O mice were singly or group-housed. It is possible that heightened anxiety-related behaviour in the 39,X^Y*^O mice could account for their locomotor hyper-reactivity to a novel homecage environment.

The pharmacological study indicated that acute inhibition of the steroid sulfatase enzyme did not recapitulate the effects of *Sts* gene deficiency on activity and anxiety-related behaviour. Whilst this finding suggests that steroid sulfatase may exert its influence on these phenotypes via neurodevelopmental pathways rather than via ongoing effects on neurosteroid metabolism, as COUMATE does not completely inhibit steroid sulfatase activity, the latter possibility cannot be completely discounted. We previously showed that acute pharmacological manipulations targeting the steroid sulfatase axis could influence aspects of attention in mice, and therefore suggested that the enzyme's ongoing activity was important in this cognitive function (Davies et al., 2009). Overall, our results indicate that steroid sulfatase may have temporally dissociable effects on the distinct brain substrates underlying attention and activity/anxiety.

We did not find any difference between 39,X^Y*^O and 40,XY mice in social dominance behaviour as assayed by the tube test; hence, steroid sulfatase is unlikely to affect this behavioural function. We previously reported that 39,X^Y*^O mice generally appeared more overtly aggressive than their 40,XY counterparts (Davies et al., 2009); in the present experimental cohort, a similar pattern of effects was observed (Supplementary Table 1). Genetic data have implicated steroid sulfatase in murine attack behaviours (Le Roy et al., 1999, Mortaud et al., 2010) whilst acute inhibition of the enzyme enhances aggression in mice (Nicolas et al., 2001). Therefore, it may be worth following up these preliminary descriptive findings in more quantitative analyses of intra-species aggression (Roubertoux et al., 2005).

Humans with steroid sulfatase deficiency are at significantly increased risk of developing inattentive subtype ADHD (and possibly autism) relative to the general population (Kent et al., 2008); a number of case studies have also reported ADHD and autistic symptoms in steroid sulfatase-deficient individuals (Thomas et al., 1999, Tobias et al., 2001, Doherty et al., 2003). Our mouse data suggest that future behavioural studies on these individuals should focus on activity and anxiety phenotypes. Following our characterisation of attention deficits in 39,X^Y*^O mice (Davies et al., 2009), we subsequently identified a single nucleotide polymorphism (rs17268988) within *STS* which was associated with inattentive symptoms in ADHD boys (Stergiakouli et al., 2011). By analogy, the present data suggest that *STS* polymorphisms may be associated with activity, drinking behaviour and anxiety endophenotypes in ADHD and autistic subjects.

Behavioural abnormalities in 39,X^Y*^O mice presumably arise due to the altered development/function of brain regions where *Sts* is expressed (together with their associated circuitry). In the murine brain, *Sts* is most highly expressed in the cortex, hindbrain and thalamus (Compagnone et al., 1997). We previously suggested that the attentional deficits observed in 39,X^Y*^O mice could be due to altered function of the pontine/basal forebrain cholinergic systems and/or to perturbed GABAergic function in the subthalamic nucleus (Davies et al., 2009). Rodents with lesions affecting the basal forebrain cholinergic system exhibit increased activity (Torres et al., 1994, van der Staay et al., 2006, Moreau et al., 2008), and heightened aggression (Bergvall et al., 1996), whilst both subthalamic nucleus lesions (Baunez et al., 2002) and infusion of the GABA_A_ receptor agonist muscimol into this nucleus (Williams and Herberg, 1987) induce hyperactivity in rodents. Examination of these neuroanatomical systems in 39,X^Y*^O mice and steroid sulfatase-deficient humans may be informative with a view to understanding how steroid sulfatase dysfunction might impact upon ADHD-relevant endophenotypes.

In conclusion, we have shown that the 39,X^Y*^O mouse, which has some degree of construct validity as an ADHD model, also exhibits a reasonable degree of face validity (at least for a subset of individuals): notably inattention, hyperactivity, heightened anxiety-related behaviours, increased water consumption and lowered systemic levels of DHEA but not corticosterone/cortisol. Investigations in the 39,X^Y*^O mouse are therefore likely to elucidate the neurobiological underpinnings of several important behavioural endophenotypes associated with ADHD (Gainetdinov, 2010).

## Role of funding sources

The funding bodies had no role in study design; in the collection, analysis and interpretation of data; in the writing of the report; and in the decision to submit the paper for publication.

## Conflict of interest

None declared.
