# Supplementary material for: Steroid sulfatase-deficient mice exhibit endophenotypes relevant to Attention Deficit Hyperactivity Disorder
Source: Psychoneuroendocrinology. 2012 Feb;37(2):221–9. doi: 10.1016/j.psyneuen.2011.06.006 (PMC3242075; doi:10.1016/j.psyneuen.2011.06.006)
Supplement: Supplementary file 1 [file mmc1.doc]

**Supplementary Material**

**Figure S1A.** Phenotyper homecage apparatus. **(A)** Top unit containing infra-red camera **(B)** Shelter **(C)** Drinking bottle connected to lickometer **(D)** Food hopper **(E)** Running wheel

**Figure S1B.** Plan view of the Phenotyper homecage showing the zones used for data analysis. **(A)** Shelter **(B)** Drinking zone **(C)** Feeding zone **(D)** Running wheel. **(E)** Arena floor. All zones combined comprised the tracking arena.

**Figure S2.** Lateral homecage activity in singly (n = 12) and group (n = 7) housed 39,X^Y*^O mice. There was no significant effect of HOUSING CONDITION on this measure (F[1,17] = 0.16, p = 0.70), nor any interaction between HOUSING CONDITION and TIMEPOINT (F[3.36,17] = 1.37, p = 0.26).

***Additional steroid hormone analysis***

Trunk blood was obtained between 12:00-14:00hrs from a subset of the 39,X^Y*^O mice for which activity data are presented (n = 7, aged ~14 months); serum samples were obtained and analysed for DHEA levels as described in the main text. DHEA levels were subsequently correlated with aggregate activity values from the homecage analysis (total distance moved (m) + total number of running wheel revolutions). This analysis indicated a similar concentration for systemic DHEA levels in 39,X^Y*^O mice as reported in the main text (0.28 ± 0.05ng/ml).

**Figure S3.** Whilst there was no strong evidence for a linear relationship between DHEA levels in 39,X^Y*^O mice and activity (Pearson correlation coefficient = -0.564, two-tailed p = 0.19), we observed that the mouse with a very low systemic DHEA concentration was the most active.

**Table S1.** Numbers of mice displaying overt aggression (as defined by consistent tail-rattling, upright posing, and attempted biting) towards the experimenter during initial handling as a proportion of total mice tested.

|  | **Experimental cohort** | |
| --- | --- | --- |
| **Genotype** | Davies et al. (2009) | Present experiment |
| 40,XY | 0/20 | 0/36 |
| 39,X^Y*^O | 2/14 | 1/19 |
|  | Fisher Exact Test, p<0.05 | |

All three 39,X^Y*^O mice showing remarkably high levels of aggression were singly-housed. In general, 39,X^Y*^O mice tended to make more audible vocalisations upon being handled, and occasionally members of this group (but not the 40,XY group) would attempt to bite the researchers.

Davies, W., Humby, T., Kong, W., Otter, T., Burgoyne, P. S., Wilkinson, L. S., 2009. Converging pharmacological and genetic evidence indicates a role for steroid sulfatase in attention. Biol. Psychiatry. 66, 360-367.
